# Zoonotic Transmission of Toxigenic *Corynebacterium ulcerans* Strain, Germany, 2012

**DOI:** 10.3201/eid2102.141160

**Published:** 2015-02

**Authors:** Dominik M. Meinel, Regina Konrad, Anja Berger, Christina König, Torsten Schmidt-Wieland, Michael Hogardt, Heribert Bischoff, Nikolaus Ackermann, Stefan Hörmansdorfer, Stefan Krebs, Helmut Blum, Gabriele Margos, Andreas Sing

**Affiliations:** Bavarian Health and Food Safety Authority, Oberschleißheim, Germany (D.M. Meinel, R. Konrad, A. Berger, C. König, M. Hogardt, H. Bischoff, N. Ackermann, S. Hörmansdorfer, G. Margos, A. Sing);; National Consiliary Laboratory for Diphtheria, Oberschleißheim (R. Konrad, A. Berger, C. König, A. Sing);; Labor Dr. Gaertner, Ravensburg, Germany (T. Schmidt-Wieland);; Institut für Medizinische Mikrobiologie und Krankenhaushygiene, Klinikum der Goethe-Universität, Frankfurt/Main, Germany (M. Hogardt);; Genecenter, Ludwig-Maximilians-University Munich, Munich, Germany (S. Krebs, H. Blum)

**Keywords:** Corynebacterium ulcerans, corynebacterium diphtheriae, diphtheria, toxigenic, zoonoses, high-throughput nucleotide sequencing

## Abstract

Severe necrotizing fasciitis was diagnosed in a 53-year-old man in Germany in 2012. Toxigenic *Corynebacterium ulcerans* was grown from a wound swab sample. One of the patient´s 2 dogs was found to harbor a toxigenic *C. ulcerans* strain. Results of next generation sequencing of both isolates supported recent zoonotic transmission of this bacterial pathogen.

Toxigenic *Corynebacterium* spp. *C. diphtheriae*, *C. ulcerans,* and rarely, *C. pseudotuberculosis* produce diphtheria toxin (DT) and thus cause respiratory and cutaneous diphtheria. DT is encoded by the phage-located *tox* gene. During the past decade, diphtheria-like infections with toxigenic *C. ulcerans* have outnumbered those caused by toxigenic *C. diphtheriae* in many industrialized countries (*1*). *C. ulcerans* has increasingly been isolated from domestic animals such as pet dogs and cats (*1*–*5*). Isolation of an undistinguishable toxigenic *C. ulcerans* strain from an animal and its owner has been documented for 2 dogs (*2*,*3*), 2 cats (*4*,*5*), and 1 pig (*6*) and their respective owners. Strain comparison was achieved in these cases by ribotyping alone (*2*,*3*), ribotyping in combination with multilocus sequence typing (*4*,*6*), or pulsed-field gel electrophoresis analysis (*5*). We report the first use of next generation sequencing (NGS) for proving zoonotic transmission of a toxigenic *C. ulcerans* strain between a pet dog and his human owner.

## The Study

In October 2012, a 53-year-old man in Baden-Wuerttemberg, Germany, whose only known underlying condition was chronic venous insufficiency, sought treatment from a private physician, who diagnosed severe necrotizing fasciitis in the calves of both legs. The patient reported no trauma, and gave no history of recent travel abroad or contact with livestock animals. The patient lived alone with his 2 dogs and had no other close personal contacts. The patient´s vaccination status against diphtheria was unknown. A swab sample from the wound on his right leg grew *Staphylococcus aureus*, *Bacteroides* spp., and *Corynebacterium ulcerans,* which were identified by biochemical differentiation (API Coryne code 0111326), including a positive O129 sensitivity test result, *rpoB* sequencing, and a score of 2.463 from Matrix-assisted laser desorption/ionisation time-of-flight analysis (MALDI Biotyper; Bruker Daltonics, Bremen, Germany). The isolate was identified as toxigenic by a positive real-time PCR for the DT-encoding *tox* gene (*7*) and a positive cytotoxicity assay by using Vero cells (toxigenic titer: 1.8; toxigenic titer of the highly toxigenic *C. diphtheriae* control NCTC 10648: 1:2000) as reported by Sing et al (*8*). However, a modified Elek test yielded a negative result (*8*). The patient was isolated at home, where he was treated with penicillin and clindamycin for 14 days and received surgical wound debridement several times over a period of 4 weeks, after which he recovered completely. The patient´s isolation at home was discontinued after 2 wound swab samples obtained 2 weeks after treatment ended showed no growth of *C. ulcerans*.

The local health authority started a comprehensive source investigation. No close human contacts could be identified. Because of known zoonotic transmission of *C. ulcerans* to humans, however, nasal and pharyngeal swab samples from the patient´s 2 asymptomatic pet dogs were obtained by the local veterinary authority. Cultures of nasal and pharyngeal swab samples from 1 of his 2 dogs grew toxigenic *C. ulcerans* and showed an API Coryne code identical to that of the human isolate. The other dog´s cultures grew several species of normal canine bacterial flora. Antimicrobial susceptibility of the isolates was tested on Mueller-Hinton blood agar (supplemented with 5% sheep blood) after overnight incubation at 37°C and 5% CO_2_. In the absence of standardized breakpoints for *C. ulcerans*, antibiotic susceptibility was determined by using the CLSI criteria for broth microbouillon dilution susceptibility testing for *Corynebacterium* species (*9*).

Both the human and the dog *C. ulcerans* strains were susceptible to amoxicillin, benzyl penicillin, ceftriaxone, erythromycin, ciprofloxacin, vancomycin, linezolid, and tetracycline, and showed intermediate susceptibility to clindamycin. Although currently no definite recommendations exist regarding antibiotic treatment of animals infected or colonized with *C. ulcerans* for preventing possible animal-to-human transmission of diphtheria-associated *Corynebacterium* spp. infection, antibiotic treatment was discussed as an option, but refused by the dog owner.

Because sequencing of *rpoB* and *tox* yielded 100% homology between the strains isolated from human and dog samples and multilocus sequence typing (*4*) suggested the clonal identity of both isolates, we aimed to confirm these findings by NGS. For a detailed genomic characterization of both isolates, we performed genome-wide resequencing on an Illumina MiSeq instrument (Illumina, Eindhoven, the Netherlands). Using Burrows-Wheeler Aligner software (SourceForge.net, Dice Holdings, New York, New York, USA), we mapped readings of both isolates to the reference genome *C. ulcerans 809* (*10*) and used VarScan (http://varscan.sourceforge.net/) for single nucleotide polymorphism (SNPs) verification. SNPs found in both isolates were discarded from the SNP list (*≈*20,000 SNPs compared to *C. ulcerans 809*). Next, we discarded the SNPs that were only found in 1 of the isolates because of technically missing sequence coverage in the other isolate data. We found that the 2 isolates did not differ by any SNPs throughout the 2.5 MB genome, clearly showing the clonal identity of both isolates. The absence of SNPs between the 2 isolates suggests a recent zoonotic transmission, because we would expect the isolates to accumulate SNPs if the transmission happened long before the strains were isolated (*11*). Furthermore, we did not detect any additional gene acquisition or genomic reordering, such as inversion or transposition in 1 of the 2 isolates but not the other. Additionally, we analyzed DT loci of both isolates and found that the DT gene is encoded by a toxigenic prophage, which is almost identical to the toxigenic phage of *C. ulcerans 0102* isolate as shown by Sekizuka et al. (*12*) in Japan.

## Conclusions

Our finding of a transmission of toxigenic *C. ulcerans* between a dog and his human owner proven by NGS underlines both the usefulness of this novel technology in a zoonotic setting of potentially toxigenic *Corynebacterium* spp. and the zoonotic potential of this organism. Previously, the proof of zoonotic transmission of *C. ulcerans* mainly had to rely on less reproducible or standardized typing methods which are more dependent on subjective interpretation, e.g., ribotyping (*2*–*4*,*6*) or pulsed-field gel electrophoresis (*5*), or on the epidemiologic circumstances in the case of deep cutaneous diphtheria manifestation caused by toxigenic *C. ulcerans* that was obviously transmitted by a cat bite (*13*). To our knowledge, the only previous use of NGS for proving zoonotic transmission of a bacterial pathogen by analyzing epidemiologically linked human and animal isolates was reported in a suspected MRSA outbreak investigation involving 2 farms where 2 separate outbreaks with sheep-to-human and cow-to-human transmission were detected by using NGS (*14*).

We found the toxigenic strain involved in the case-patient´s skin ulceration to be *C. ulcerans*–negative using a modified Elek test, but positive in a cytotoxicity assay using Vero cells. Similar findings have been reported previously for toxigenic *C. ulcerans*, suggesting a higher sensitivity of cytotoxicity assays than that seen with the Elek test (*15*). Because DT production by toxigenic *C. ulcerans* is reported to be substantially lower than that usually seen in toxigenic *C. diphtheriae* (*8*) as was the case in the study patient, it is difficult to estimate the pathogenic contribution of these low amounts of DT detected only in vitro by using a cytotoxicity assay, but not by immunologic precipitation for this patient in vivo. Furthermore, as described first for *C. diphtheriae*, also nontoxigenic *tox*-bearing strains of *C. ulcerans* exist, and some of them originate in animals (*8*).

In conclusion, the declining costs of NGS and the increasing availability of bioinformatics tools should make this method more available. The wider introduction of this technology in public health laboratories will help in outbreak investigations and support public health authorities in the management of infectious diseases.
